# 山东地区非小细胞肺癌分子靶向治疗驱动基因表达情况及临床特征分析

**DOI:** 10.3779/j.issn.1009-3419.2017.01.02

**Published:** 2017-01-20

**Authors:** 秀丽 乔, 丹 艾, 洪陆 梁, 殿斌 穆, 其森 郭

**Affiliations:** 1 50200 济南，济南大学 山东省医学科学院医学与生命科学学院 School of Medicine and Life Sciences, University of Jinan-Shandong Academy of Medical Sciences, Jinan 250200, China; 2 250117 济南，山东省肿瘤医院，山东省医学科学院 Shandong Tumor Combat Research Institute, Jinan 250117, China

**Keywords:** 肺肿瘤, 分子靶向治疗, 表皮生长因子受体, 棘皮动物微管相关蛋白4-间变性淋巴瘤激酶, 肉瘤致癌因子-受体酪氨酸激酶, 鼠类肉瘤病毒癌基因, Lung neoplasms, Molecular target therapy, Epidermal growth factor receptor, Echinoderm microtubule-associated protein-like 4-anaplastic lymphoma kinase, ROS proto-oncogene 1, Kirsten rat sarcoma viral oncogene

## Abstract

**背景与目的:**

分子生物学靶向治疗已逐渐成为非小细胞肺癌（non-small cell lung cancer, NSCLC）的一个重要治疗手段，本研究通过分析山东地区NSCLC多种驱动基因表达情况及临床病理特征，为筛选分子靶向治疗目标人群提供理论依据。

**方法:**

采用荧光探针PCR法检测表皮生长因子受体（epidermal growth factor receptor, *EGFR*）、棘皮动物微管相关蛋白4-间变性淋巴瘤激酶（echinoderm microtubule-associated protein-like 4-anaplastic lymphoma kinase, *EML4*-*ALK*）、肉瘤致癌因子-受体酪氨酸激酶（ROS proto-oncogene 1, receptor tyrosine kinase, *ROS1*）、鼠类肉瘤病毒癌基因（Kirsten rat sarcoma viral oncgene, *KRAS*）基因表达情况，回顾性分析阳性病例的临床病理特征。

**结果:**

*EGFR*基因突变阳性率为36.70%，主要为19、21外显子突变，突变人群主要为女性、腺癌、不吸烟患者，组间差异有统计学意义。*EML4*-*ALK*融合基因重排阳性率为9.37%。人群特征主要为60岁以下不吸烟人群，组间差异有统计学意义，基因突变与病理类型和性别间无明显差异。*ROS1*融合基因重排阳性率为3.67%，均为60岁以下患者，组间差异有统计学意义。23份病例标本开展*KRAS*基因检测，阳性标本数2例，阳性率为8.70%。2份阳性标本均为60岁以上病例，男女各占1例，病理类型均为腺癌，均无吸烟史。此外，未发现有两种基因同时突变的病例。

**结论:**

*EGFR*、*EML4*-*ALK*、*ROS1*、*KARS*基因在NSCLC患者中存在较高的突变率，且具有不同的人群特征，在选择靶向治疗人群中具有重要意义。

## Abstract (en)

**Background and objective:**

Molecular targeted therapy has gradually become an important treatment for lung cancer, the aim of this research is to analyze the clinicopathologic features associated with the gene mutation status of epidermal growth factor receptor (*EGFR*), echinoderm microtubule-associated protein-like 4-anaplastic lymphoma kinase (*EML4*-*ALK*), ROS proto-oncogene 1, receptor tyrosine kinase (*ROS1*) and Kirsten rat sarcoma viral oncogene (*KRAS*) in non-small cell lung cancer (NSCLC) patients and determine the most likely populations to benefit from molecular target therapy treatment.

**Methods:**

The mutation status of *EGFR*, *EML4*-*ALK* fusion gene, *ROS1* and *KARS* gene were determined by Real-time PCR, the relationship between clinical pathologic features and concomitant gene were analyzed with χ^2^ test by SPSS software 19.0.

**Results:**

A total of 514 specimens from Shandong tumor hospital were collected from NSCLC patients between January 2014 and May 2016. The total mutation rate of *EGFR* gene was 36.70%, major occurred in exon 19 (36.61%) and exon 21 (51.36%), respectively, and *EGFR* mutations usually occurred in female, non-smoking and adenocarcinoma patients (*P* < 0.05). The total rearrangements rate of *EML4*-*ALK* fusion gene was 9.37%, *EML4*-*ALK* fusion gene usually occurred in younger age (≤60 yr) and non-smoking patients (*P* < 0.05). Mutations were not related to gender and pathological type (*P* > 0.05). *ROS1* fusion gene was detected in 136 cases, the positive rate was 3.67%, all patients were 60 years old, and the difference was statistically significant (*P* < 0.05). Only 23 samples were tested *KARS* gene mutations, two of them were positive and the positive rate was 8.70%. They all occurred in non-smoker and adenocarcinoma patients. No mutation was detected to coexist in *EGFR*, *EML4*-*ALK* and *KARS* gene mutation.

**Conclusion:**

EGFR, *EML4*-*ALK*, *ROS1* and *KRAS* defines different molecular subset of NSCLC with distinct characteristic, which provides a new option for the clinical treatment of patients with NSCLC.

肺癌可分为小细胞癌和非小细胞癌（non-small cell lung cancer, NSCLC）等，其中NSCLC约占80%-85%，传统的化疗和放疗缺乏特异性，副作用较大，大约66%的NSCLC患者在确诊时已是晚期，总体预后较差。2004年-2006年，表皮生长因子受体（epidermal growth factor receptor, *EGFR*）基因作为新发现的NSCLC驱动基因，引起全球肿瘤学领域学者的关注^[1, 2]^。随着人类基因组学和蛋白组学突破性的进步，目前在肺癌中发现10余种驱动基因，这些驱动基因的检测为肺癌的治疗和生存期的延长提供重要帮助。2014年《非小细胞肺癌临床实践指南》除了继续推荐对于复发或转移的NSCLC患者进行*EGFR*突变和间变性淋巴瘤激酶（anaplastic lymphoma kinase, *ALK*）重排检测外，更加强调治疗初期的多重癌基因检测。本研究通过检测山东地区NSCLC患者的*EGFR*、棘皮动物微管相关蛋白4-ALK（echinoderm microtubule-associated protein-ALK, *EML4*-*ALK*）、肉瘤致癌因子-受体酪氨酸激酶（ROS proto-oncogene 1, receptor tyrosine kinase, *ROS1*）、鼠类肉瘤病毒癌基因（Kirsten rat sarcoma viral oncogene, *KRAS*）基因表达情况，分析阳性标本的临床病理特征，为筛选分子靶向治疗特异人群提供数据支持。

## 1 资料和方法

### 1.1 标本来源

选取2014年1月-2016年5月期间山东省肿瘤医院确诊的NSCLC患者的石蜡标本，完善临床信息，包括：年龄、性别、病理类型、吸烟史等。所有标本均为通过纤维支气管镜或计算机断层扫描（computed tomography, CT）引导下肺肿瘤穿刺并病理检测诊断为NSCLC病例标本。

### 1.2 检测方法

石蜡标本RNA提取试剂盒和DNA提取试剂盒均为美国QIAGEN公司产品，*EGFR*基因29种突变检测试剂盒（荧光探针PCR），*EML4*-*ALK*融合基因检测试剂盒（荧光探针PCR)，*ROS1*融合基因检测试剂盒（两步RT-PCR法）、人类*K*-*ras*基因7种突变检测试剂盒（荧光探针PCR）均采用厦门艾德生物医药科技有限公司产品，具体操作按照试剂盒说明书进行。

### 1.3 统计学方法

采用SPSS 19.0软件进行统计学分析，定性资料采用卡方检验或精确概率法，以*P*＜0.05为差异有统计学意义（双侧检验）。

## 2 结果

### 2.1 基本情况

共收集514例NSCLC病例标本，其中2014年病例117例，2015年255例，2016年142例。最小年龄30岁，最大年龄85岁，平均年龄（59.68±11.19）岁。男性306例，女性208例，男女性别比为1.47:1。按照世界卫生组织（World Health Organization, WHO）肺癌组织分类标准进行组织分型，腺癌为422例，鳞癌为72例，其他为20例（腺鳞癌、大细胞肺癌、肉瘤样癌）。吸烟人数300例，不吸烟人数为214例，所有患者术前均未接受过化放疗。

### 2.2 *EGFR*基因突变情况及其与临床特征的关系

共有466例病例开展*EGFR*基因检测，其中突变样本数171例，阳性率为36.70%，其中18外显子突变2例，突变率为0.43%。19外显子突变63例，突变率为13.52%，20外显子突变6例，突变率为1.28%，21外显子突变88例，突变率为18.88%。双基因突变12例，突变率为2.58%。171例阳性标本中，21外显子L858R突变所占比例最高为50.27%，其次为19外显子缺失突变，占总突变数的36.61%，20外显子T790M突变占突变总数的7.10%，其他类型所占比例较低，详见表 1。此外，发现12例两个位点同时突变的病例，其中4例19外显子缺失突变合并20外显子T790M位点突变，5例21外显子L858R位点突变合并20外显子T790M位点突变。2例18外显子G719X合并20外显子S768I位点突变，1例18外显子G719X合并21外显子L861Q位点突变。

**1:** 山东地区NSCLC病例*EGFR*基因突变类型分析 Characteristic of *EGFR* gene mutation types in 466 NSCLC patients

*EGFR* mutation type	Positive number	Proportion (%)
18 exon (G719X)	5	2.73
19 exon (-del)	67	36.61
20 exon (T790M)	13	7.10
20 exon (S768I)	4	2.19
21 exon (L858R)	92	50.27
21 exon (L861Q)	2	1.09
Total	183^§^	100.00
NSCLC: non-small cell lung cancer; EGFR: epidermal growth factor receptor; §: include 159 single site mutation patients and 12 double locus mutation patients.		

≤60岁年龄组*EGFR*基因突变率略高于60岁以上年龄组人群，经统计学分析，组间差异无统计学意义（χ^2^=0.86, *P*＜0.05）。女性*EGFR*基因突变率显著突变率高于男性，且差异有统计学意义（χ^2^=21.42, *P*＜0.05）。腺癌*EGFR*基因突变率显著高于其他病理类型，差异有统计学意义（χ^2^=22.86, *P*＜0.05）。不吸烟人群*EGFR*基因突变率显著高于吸烟人群，且差异有统计学意义（χ^2^=19.93, *P*＜0.05）。详见表 2。

**2:** 山东地区NSCLC患者*EGFR*基因突变与临床特征的关系 Correlation between *EGFR* gene mutation and clinical features in 466 NSCLC patients

Group (EGFR)	Number of detected	Number of positive	Positive rate (%)	*χ*^2^	*P* value
Age (yr)				0.83	0.86
≤60	246	95	38.62		
＞60	220	76	34.55		
Gender				21.42	＜0.001
Male	277	78	28.16		
Female	189	93	49.21		
Histology				22.86	＜0.001
Ad	402	165	41.04		
SCC	51	5	9.80		
Others	13	1	7.69		
Smoking status				19.93	＜0.001
Yes	280	80	28.57		
No	186	91	48.92		
Total	466	171	36.70		
Ad: adenocarcinoma; SCC: squamous cell carcinoma.					

### 2.3 *EML4*-*ALK*融合基因表达情况及与临床特征的关系

共491例病例开展*EML4*-*ALK*融合基因检测，融合基因阳性率为9.37%。60岁以下年龄组*EML4*-*ALK*融合基因重排阳性率明显高于60岁以上年龄组，组间差异有统计学意义（χ^2^=14.10, *P*＜0.05）。女性*EML4*-*ALK*融合基因重排阳性率高于男性，组间差异无统计学意义（χ^2^=2.75, *P*＞0.05）。腺癌的*EML4*-*ALK*融合基因重排阳性率略高于鳞癌，组间差异无统计学意义（χ^2^=1.38, *P*＞0.05）。不吸烟人群的*EML4*-*ALK*融合基因重排阳性率显著高于吸烟人群，组间差异有统计学意义（χ^2^=4.42, *P*＜0.05）。详见表 3。

**3:** 山东地区NSCLC病例*EML4*-*ALK*融合基因重排情况及其与临床特征的关系 Correlation between *EML4*-*ALK* fusion gene rearrangements and clinical features in 491 NSCLC patients

Group (*EML4*-*ALK*)	Number of detected	Number of positive	Positive rate (%)	*χ*^2^	*P* value
Age (yr)				14.10	＜0.001
≤60	266	37	13.91		
＞60	225	9	4.00		
Gender				2.75	0.097
Male	291	22	7.56		
Female	200	24	12.00		
Histology				1.38	0.51
Ad	426	42	9.86		
SCC	56	4	7.14		
Others	9	0	0		
Smoking status				4.42	0.035
Yes	285	20	7.02		
No	206	26	12.62		
Total	491	46	9.37		
EML4-ALK: echinoderm microtubule-associated protein-like 4-anaplastic lymphoma kinase.					

### 2.4 *ROS1*融合基因表达及与临床特征的关系

共136例病例开展*ROS1*融合基因检测，重排阳性率为3.68%，60岁以下年龄组*ROS1*融合基因重排阳性率明显高于60岁以上年龄组，组间差异有统计学意义（χ^2^=0.032, *P*＜0.05）。未发现该基因阳性与性别、吸烟史和组织类型有明显关联，组间差异无统计学意义，详见表 4。

**4:** 山东地区NSCLC病例*ROS1*融合基因重排情况及其与临床特征的关系 Correlation between *ROS1* gene rearrangements and clinical features in 136 NSCLC patients

Group（ROS1）	Number of detected	Number of positive	Positive rate (%)	*χ*^2^	*P* value
Age				4.61	0.032
≤60	72	5	6.94		
＞60	64	0	0		
Gender				0	0.98
Male	81	3	3.70		
Female	55	2	3.64		
Histology				0.64	0.73
Ad	121	5	4.13		
SCC	10	0	0		
Others	5	0	0		
Smoking status				0.015	0.90
Yes	78	3	3.85		
No	58	2	3.45		
Total	136	5	3.68		
Ad: adenocarcinoma; SCC: squamous cell carcinoma.					

### 2.5 *KRAS*基因表达与临床特征的关系

23份病例标本开展*KRAS*基因检测，阳性标本数2例，阳性率为8.70%。2份阳性标本均为60岁以上病例，男女各占1例，病理类型均为腺癌，均无吸烟史。

### 2.6 *EGFR*、*EML4*-*ALK*、*ROS1*、*KRAS*基因关联性分析

454例病例开展两种及以上基因突变情况检测，均未发现有两种基因同时阳性的病例。

## 3 讨论

EGFR是目前最主要的分子治疗靶点，有文献报道的*EGFR*酪氨酸激酶结构域的突变已达30余种，目前具有靶向治疗意义的大致分为3类：19外显子的缺失、18和21外显子的单核苷酸的替换突变及20外显子的复制突变，其中21外显子的L858R和19外显子的缺失突变占绝大多数。不同地域*EGFR*基因突变种类不尽相同，如我国昆明地区*EGFR*基因突变全部发现在19外显子，而江浙地区患者*EGFR*基因突变主要为19和21外显子^[3-6]^。本研究显示山东地区*EGFR*基因突变率为36.70%，突变主要为19和21外显子突变，合计占突变总数的87.97%；突变人群主要为女性、腺癌、不吸烟患者与既往研究一致，与江浙地区人群一致。

EGFR受体抑制剂包括小分子酪氨酸激酶抑制剂（tyrosine kinase inhibitor, TKI）和单克隆抗体。研究^[7]^显示并不是所有的患者都能从EGFR-TKI中获益，易瑞沙肺癌生存评估（The IRESSA Survival Evaluation in Lung Cancer, ISEL）研究发现，女性、不吸烟者、腺癌及东亚人群从EGFR-TKI中获益最大。2004年，Lynch^[2]^和Paez^[1]^等首次提出，EGFR-TKI对不同NSCLC的治疗效果的差异是由于EGFR外显子的突变状态不同。EGFR19或/和21外显子的突变异常将导致EGFR结合ATP的能力增加，从而加强了TKI的疗效，而20外显子突变可使肿瘤细胞对EGFR-TKI产生抵抗性^[8-10]^。本研究发现山东地区20外显子T790M和S768I突变分别占到突变总数的7.10%和2.19%，12例两个位点同时突变的病例中4例19外显子缺失突变合并20外显子T790M位点突变，5例21外显子L858R位点突变合并20外显子T790M位点突变。在开展分子靶向治疗人群选择时应引起重视。

既往研究^[11-14]^显示，*EML4*-*ALK*融合基因在东亚地区肺癌患者中发生率为3%-7%，且更易表达于肺腺癌这一组织学亚型中。我国林小梅^[15]^等研究*EML4*-*ALK*融合基因阳性率为7.8%，多出现在小年龄、女性、不吸烟患者中。钟山等^[16]^研究268例NSCLC患者中*EML4*-*ALK*融合基因重排阳性率为4.1%，多出现在≤60岁、女性、不吸烟患者中，但组间差异无统计学意义。朱翔等^[17]^研究发现525例肺癌患者中ALK免疫组化阳性率为5.14%，多出现在年轻患者、女性、肺腺癌中，组间差异有统计学意义。潘丽霞等^[18]^研究252例NSCLC患者*EML4*-*ALK*融合基因重排阳性率为4.7%，基因突变多为女性、年龄较小者（≤60岁），与吸烟史和病理类型无关^[19]^。本研究发现山东NSCLC患者*EML4*-*ALK*融合基因重排阳性率为9.37%，主要为60岁以下不吸烟人群，病理类型和性别间无明显差异，与既往研究略有差异，需要进一步分析。

ROS1属于酪氨酸激酶胰岛素受体基因，在NSCLC中，最早确认*ROS1*易位报道是在一位亚洲患者中，代表一类新的独特的NSCLC分子亚型，其发生频率为1%-2%，远低于*EGFR*突变和*ALK*重排变异，且存在一定的种族差异^[20, 21]^，但在EGFR/KRAS/ALK均阴性的人群中发生率则可明显提高到5.7%^[22]^。*ROS1*基因重排阳性与*ALK*重排阳性的NSCLC患者具有相似性的临床特征，即为年轻、从不吸烟且为高恶性度趋势的肺腺癌患者^[20-23]^。研究显示运用即时PCR法检测302例肺癌肿瘤组织样本，12例检测出*ROS1*融合基因阳性，总阳性检出率为3.97%。但是年龄、性别、吸烟、组织类型均无统计学意义^[16]^。本研究*ROS1*融合基因重排阳性率为3.68%，突变人群主要为小年龄组患者。由于*ROS1*与ALK的酪氨酸激酶区域约有49%的同源性，目前至少有4种ALK的抑制剂也可作为*ROS1*的抑制剂，因此虽然该基因突变率较低，但开展*ROS1*基因研究具有重要意义。

KRAS阳性表达会使患者的生存预后显著降低，2014年《非小细胞肺癌临床实践指南》明确指出：当*KRAS*基因发生突变时，不建议使用EGFR-TKIs靶向治疗药物。该基因点突变以第2号外显子11、12、13、59和61密码子多见，其中96%发生在第2号外显子12、13密码子。这些密码子编码的氨基酸是*KARS*蛋白与GTP酶活化蛋白（GAP）作用位点。研究显示与EGFR不同，*KRAS*突变常见于吸烟、腺癌患者，20%见于白种人，5%见于黄种人^[23-26]^。国内研究^[27]^205例EGFR阴性NSCLC患者中KARS基因突变阳性率为23.8%，多为男性、小于65岁、腺癌患者，但组间差异无统计学意义。张卉等^[28]^研究395例肺腺癌患者中*KRAS*基因突变率为7.8%，在性别、年龄、临床分期、吸烟史组间均无统计学差异。本次研究发现山东地区NSCLC病例*KRAS*基因突变率为8.70%，两例阳性突变均为G12A，均为60岁以上病例，男女各占1例，病理类型均为腺癌，均无吸烟史。由于其检测数量较少，人群特征需要进一步观察。此外，本研究未发现有两种基因同时阳性的病例，与多数研究报告相一致。虽然有研究报道*EGFR*基因和*EML4*-*ALK*融合基因同时阳性的病例，但是这种现象仍属于罕见现象^[29-31]^。

综上所述，NSCLC患者*EGFR*、*EML4*-*ALK*、*ROS1*、KARS基因突变人群具有不同的临床病理特征，对肿瘤患者进行准确的基因检测，分析临床病理特征，找到合适人群，可使治疗效果最大化。
